# Understanding the interactions of poly(methyl methacrylate) and poly(vinyl chloride) nanoparticles with BHK-21 cell line

**DOI:** 10.1038/s41598-020-80708-0

**Published:** 2021-01-22

**Authors:** Gomathi Mahadevan, Suresh Valiyaveettil

**Affiliations:** Present Address: Department of Chemistry, National University of Singapore, 3 Science Drive 3, Singapore, 117543 Singapore

**Keywords:** Biological techniques, Environmental sciences, Chemistry, Nanoscience and technology

## Abstract

Microplastic and nanoplastic particles are prevalent in the environment and are beginning to enter the living system through multiple channels. Currently, little is known about the impact of plastic nanoparticles in living organisms. In order to investigate the health impact of micro- and nanoparticles of common polymers in a systematic way, luminescent plastic nanoparticles from two common polymers, polyvinyl chloride (PVC) and poly (methyl methacrylate) (PMMA) with relatively narrow size distribution are prepared using a nanoprecipitation method. As a model system, BHK-21 cells were exposed to polymer nanoparticles to understand the mode of uptake, internalization and biochemical changes inside the cells. The cellular effects of the nanoparticles were evaluated by monitoring the changes in cell viability, cell morphology, concentrations of reactive oxygen species (ROS), adenine triphosphate (ATP) and lactate dehydrogenase at different concentrations of the nanoparticles and time of exposure. PVC and PMMA nanoparticles induced a reduction in the cell viability along with a reduction of ATP and increase of ROS concentrations in a dose- and time-dependent manner. The plastic nanoparticles are internalized into the cell via endocytosis, as confirmed by Dynasore inhibition assay and colocalization with latex beads. Our findings suggest that plastic nanoparticle internalization could perturb cellular physiology and affect cell survival under laboratory conditions.

## Introduction

Plastic waste accumulated in the environment undergoes slow degradation and disintegration under the ambient conditions and in presence of sunlight to generate smaller fragments called microplastics (size below 5 mm) or nanoplastics (below 1 µm)^1,2^. Recent studies have shown that such plastic particles present in the environment are entering the food chain and cause adverse health impacts^3^. The most commonly identified microplastic particles in the environment are poly(styrene) (PS), poly(ethylene terephthalates) (PET), polyethylene (PE), poly(propylene) (PP), poly(vinyl chloride) (PVC), poly(methyl methacrylate) (PMMA) and poly(vinyl butyral) (PVB)^4–11^. In order to understand the impact of micro- and nanoplastics on human health, research efforts are focused on understanding the cellular uptake of plastic particles in recent years^12–18^. Among the many mechanisms involved in the uptake of microplastic particles^19^, the endocytosis pathway^20^ is the prominent one. Cellular uptake of microplastics is also depends on the size^21–24^ and surface charge of the particles^25–27^.

Understanding the interaction of plastic nanoparticles (NPs) and cells helps to identify the impact of plastic pollution towards the health of living organism. PVC, PMMA, PE, PP, PET and PS are the most common polymers present in the plastic waste, therefore understanding their interactions with living organisms is important. For the current investigation, we are interested in exploring the uptake and translocation of NPs of two most common polymers, PVC and PMMA and investigated the induced cellular biochemical changes using baby hamster kidney cells (BHK—21) as a model. For easy tracking purpose, fluorescent perylene dye incorporated NPs were prepared from these two polymers using the nanoprecipitation method^28^ and fully characterized. Both mechanisms of intake of the luminescent polymer NPs and biochemical changes occurring inside the cells are discussed in detail. Unique to this investigation, a comparison on the interactions of PVC and PMMA NPs with BHK cells is given at the end of the paper.

## Results

### Preparation and characterization of plastic nanoparticles

**Figure 1:**
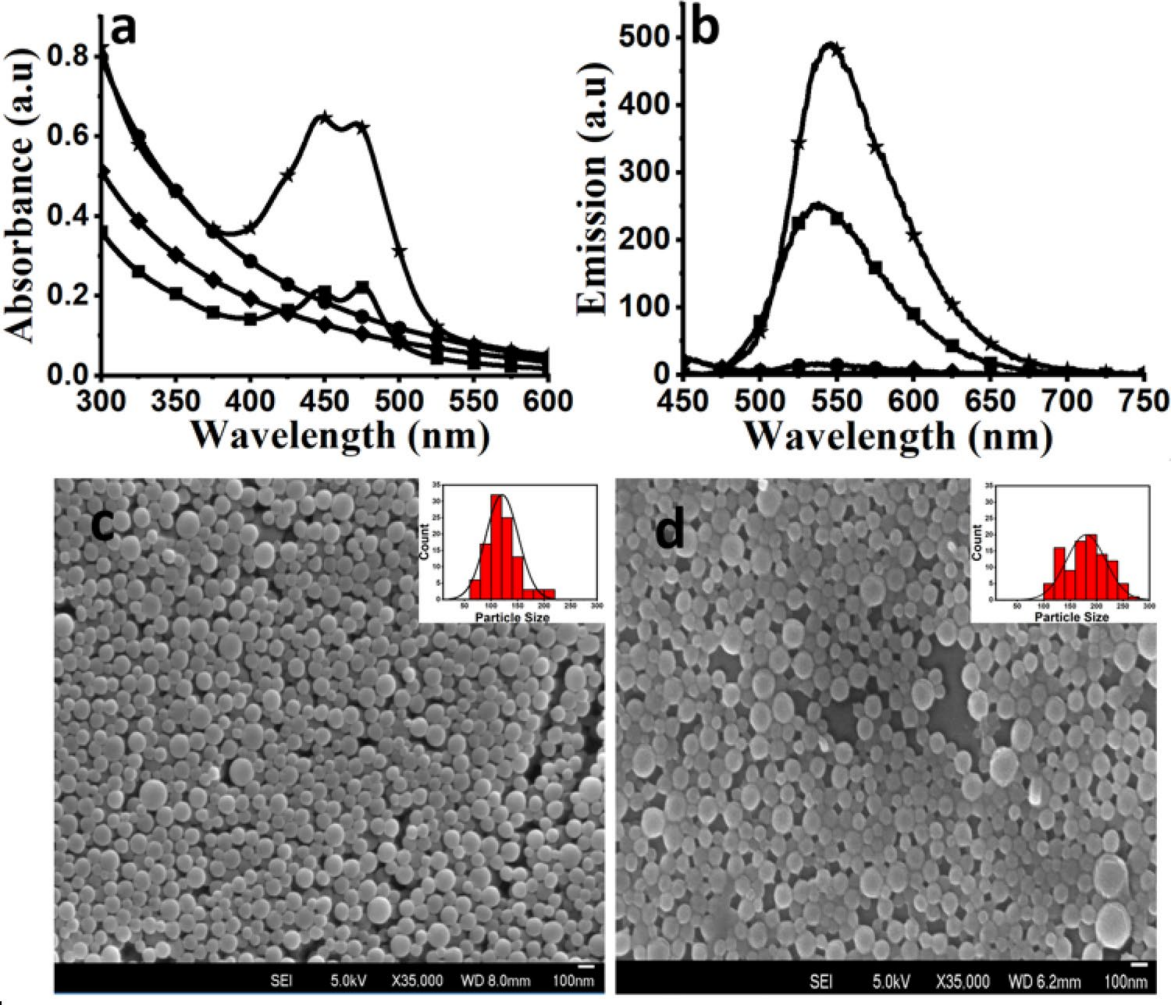
Absorption (**a**) and emission (**b**) spectra of polymer NPs, PVC (*), PMMA (■), and pure polymers, PVC (●) and PMMA (♦) solutions. The NPs are dispersed in water and spectra for pure polymer for comparison was recorded in tetrahydrofuran**.** SEM images of PMMA (c) PVC (d) NPs, insets represent the size distribution curve of polymer NPs obtained from SEM image. Scale bar − 100 nm.

Nanoparticles (NPs) of PMMA and PVC were prepared using a nanoprecipitation method^28^. By controlling the experimental parameters, stable dispersions of fluorescent polymer NPs in water were obtained. Full structural characterization and photophysical properties of the perylene tetraester (PTE) dye encapsulated within PVC and PMMA NPs were done using scanning electron microscopy, absorption and emission spectroscopy (Fig. 1a,b). The absorption maxima of the NPs were at 445 nm and 470 nm, and emission maximum was at 550 nm, respectively, which is identical to the solution spectrum of the dye^29^. From the absorbance and emission spectra, spectral inversion or bathochromic shift characteristic of dye aggregation in the NPs was not observed^28^, indicating homogenous distribution of dye inside the polymer NPs. The order of mixing of organic polymer solution with water has an impact on the NP size distribution. Adding water to the polymer solution led to the formation of larger particle aggregates and adding polymer solution to larger amounts of water gave smaller particles with a narrow size distribution.

From DLS measurements, the average hydrodynamic size of the PMMA NPs was ~ 140 ± 16 nm and that for PVC NPs was ~ 120 ± 18 nm (Table 1, Figure S1). The zeta potentials for PMMA and PVC NPs were − 26.8 mV and − 39.3 mV, respectively. The size and morphology of the polymer NPs were also obtained from scanning electron microscopy (SEM) analyses (Fig. 1c,d). The average size of monodispersed NPs calculated from SEM data was 150.0 ± 4.3 nm for PMMA (Fig. 1c, inset) and 110.0 ± 3.0 nm for PVC (Fig. 1d, inset) NPs. The standard deviation for the DLS data (i.e. hydrated radius) is usually larger than the values obtained from SEM micrograph (i.e. dry radius). Moreover, both polymers are hydrophobic in nature and therefore the difference between the DLS and SEM data is not significant. To remove the traces of impurities such as surfactants, PTE and organic solvent left in solution after the precipitation, the prepared NPs were centrifuged and washed repeatedly with sterilized deionized water. The DLS data of NPs obtained before and after washing showed that the washings did not alter the size or stability of the NPs significantly.

**Table 1:** Size and surface charges of the PMMA and PVCs NPs from DLS measurements in water before and after washing.

Polymer NPs	As synthesized		Washed and centrifuged (10,000 rpm)	
	D* (nm)	Ζ** (mV)	D* (nm)	Ζ** (mV)
PVC	120 ± 18	−39.3	125 ± 18	−26.4
PMMA	140 ± 16	−26.8	140 ± 16	−24.6

*D: Hydrodynamic diameter (nm).

**Z: Zeta-potential (mV), PDI values where 0.05 for PVC and 0.07 for PMMA NPs.

### Effects of NP-associated surfactants on cell viability

The surfactants such as sodium dodecyl sulphate (SDS) used for stabilization of the polymer nanoparticles may interfere with the cell viability^30^. The NPs were prepared using a SDS concentration of 4% for stabilization in water, but NP solution was diluted further with cell medium with a final SDS concentration of 0.4% in each well. It is important to note that all SDS are incorporated inside the polymer NPs and little left in the solution after repeated washings of the particles. As a control, a separate solution (0.4%) of SDS without any polymer NPs was prepared and added to the cells. Similarly, another solution of SDS at a high concentration (4.0%) was also used as a control to check the toxicity (Figure S2). Data in the presence of SDS at a low concentration (0.4%) did not show toxicity to the BHK-21 cells as compared to control cells with no additives, while at a higher concentration (4%) showed 40% reduction in viability.

### Effect of trace amounts of organic solvent in the particle solution

The interference from the traces of organic solvents (e.g. acetone and tetrahydrofuran) used for the particle synthesis on BHK-21 cells was also investigated. To check the solvent interference in the cytotoxicity, the organic solvent, acetone or tetrahydrofuran (5 mL) was added to water and stirred for 18 h, as per the procedure used for the polymer particle synthesis. No polymers or SDS was added in the solution and most of the organic solvents evaporate from water during this period. After stirring the solution, similar volume of the solution (10 µL) was added to the BHK-21 cells (5000 cells/ well) kept inside a 96 well plate along with media (90 µL), incubated for 24 h at 37 °C in CO_2_ (5%) atmosphere and high humidity (95%). The cells were collected and exposed to Alamar blue assay to measure the cell viability. The results showed that the cell viability was not affected by the addition of the solution which contain trace amounts of organic solvents used for the preparation of polymer NPs (Figure S3). Similarly, the impact of unreacted reagents on the cytotoxicity of PMMA and PVC NPs, appropriate volume of supernatant (10 µL) solution collected after centrifugation of the NPs was added to BHK-21 cells along with medium (1 mL). The cell viability results from the Alamar blue assay showed no evidence of toxicity induced by the supernatant solution from PVC or PMMA NPs (Figure S4).

### Cytotoxicity of PVC and PMMA NPs

**Figure 2:**
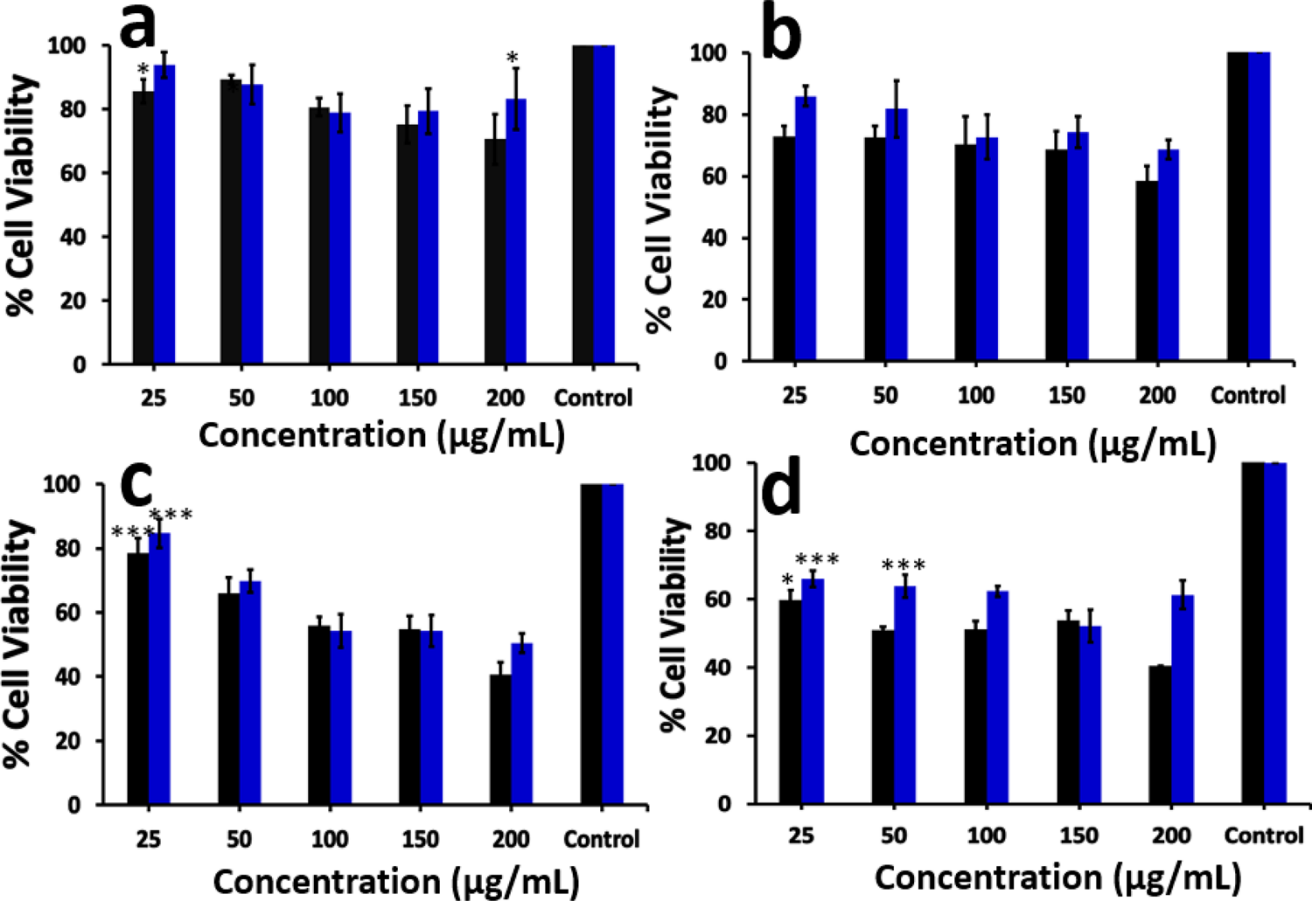
Cell viability of BHK-21 cells exposed to different concentrations of PVC (■) and PMMA ( ) NPs for 24 h (**a**), 48 h (**b**), 96 h (**c**), 120 h (**d**). The data for 72 h incubation is given in supporting information Figure S5. The results are expressed as % of the viability as compared to the control, mean ± SEM values of n = 3 independent experiments are given. Statistical analysis was done by one-way ANOVA (*p < 0.05, **p < 0.01, ***p < 0.001, ****p < 0.0001) and compared to control cells with no NP exposure.

To gauge the toxicity of PVC and PMMA NPs to BHK-21 cells, a commonly used cell line for viral titration, were exposed to the NPs and cell viability was assessed using the Alamar blue assay. Viability of BHK-21 cells was not significantly affected at lower NP concentration (25 µg/mL), whereas at high concentrations (200 µg/mL) of PVC or PMMA NPs caused a reduction in cell viability over time (Fig. 2). Cell viability was decreased when cells were exposed to increasing concentrations (25, 50, 100, 150 and 200 µg/mL) of the NPs in a time dependent manner. The purpose of using high concentrations of nanoparticle here is to observe changes in cellular activities within a short period. Cell viability was not affected (Fig. 2a) up to 24 h and decreased to 58% for PVC and 68% for PMMA at 48 h (Fig. 2b). At high NP concentration (200 µg/mL), significant reductions in viability was observed (40.3 ± 0.1% for PVC and 61.3 ± 4.0% for PMMA NPs) after 120 h (Fig. 2c,d). Similar decrease in viability was observed with increase in time to 72 h (Figure S5). This data also indicate that PVC NPs are more toxic than PMMA NPs at high concentrations and prolonged incubation.

### Cellular internalization of nanoparticles

**Figure 3:**
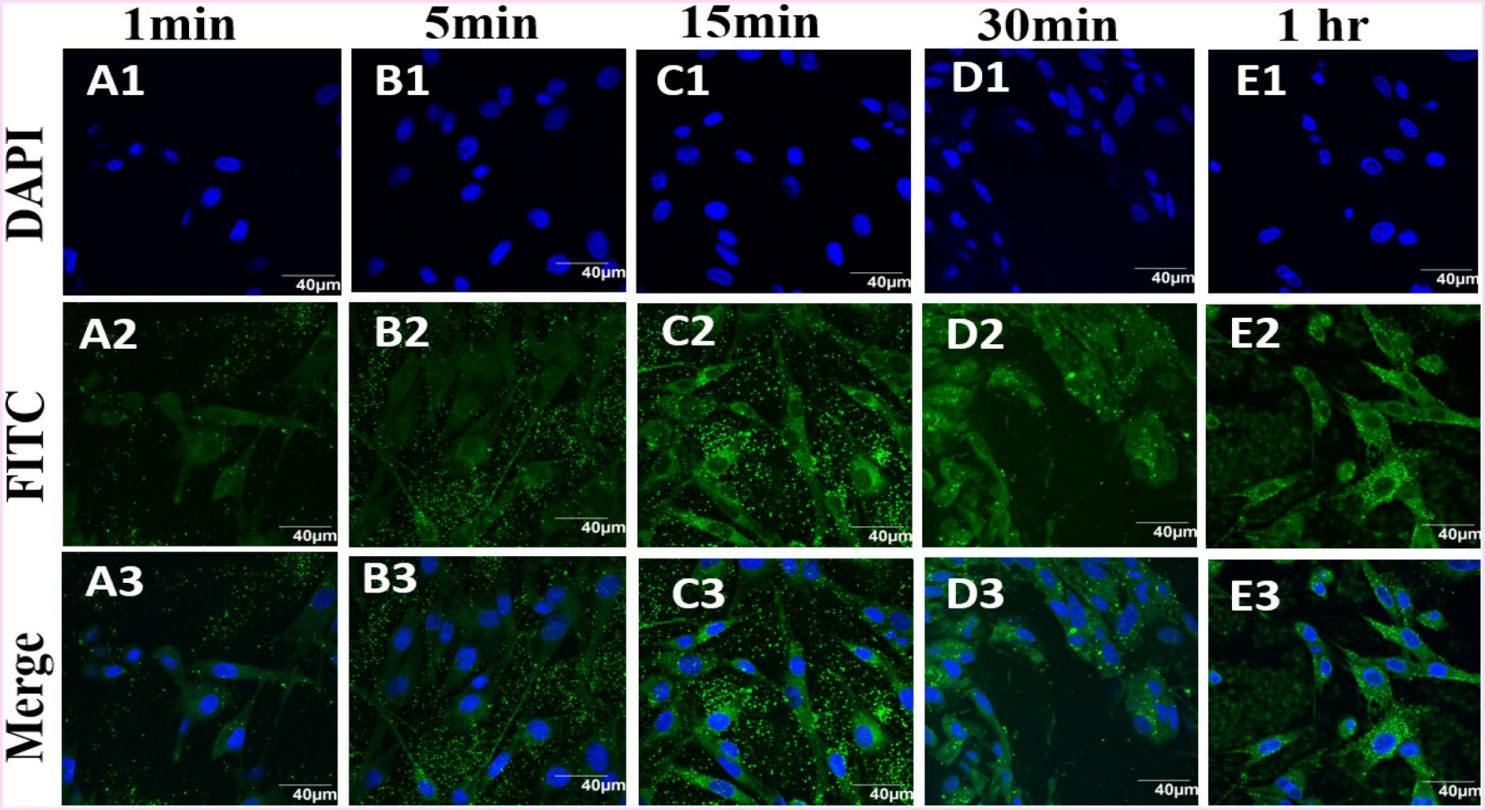
Time based tracking of PVC NPs (200 µg/mL) exposed to BHK-21 cells. Top to bottom rows: Hoechst stain in DAPI channel, NPs appear as green in FITC channel, overlay of blue and green channels. Images observed at (**A1**–**A3**) 1 min, (**B1**–**B3**) 5 min, (**C1**–**C3**) 10 min, (**D1**–**D3**) 30 min, and (**E1**–**E3**) 1 h are given to compare the time dependance internalization of PVC particles. All images were recorded using an Olympus FV1000 confocal microscope. The control experiment for all the time points are given in the supporting information, Figure S6.

**Figure 4:**
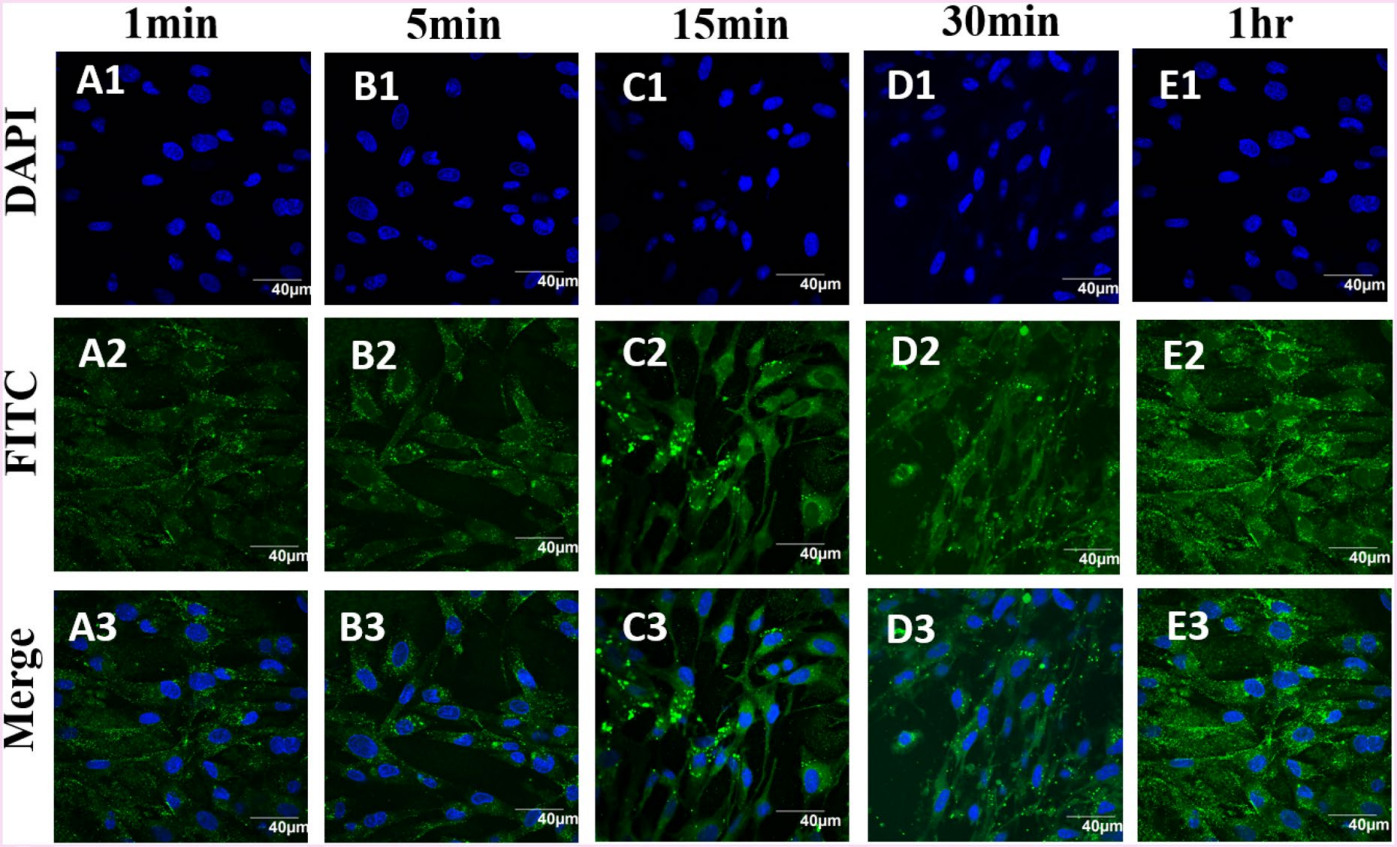
Confocal images of the BHK-21 cells exposed to PMMA nanoparticles (200 µg/mL) after 1 min (**A1**–**A3**), 5 min (**B1**–**B3**), 15 min (**C1**–**C3**), 30 min (**D1**–**D3**) and 1 h (**E1**–**E3**). From top to bottom rows: Hoechst stain in blue DAPI channel, NPs appear as green FITC channel, overlay of blue and green channels. The scale bar is 40 µm. The control experiment for all the time points are given in the supporting information Figure S6.

The cellular internalisation of NPs was investigated using confocal microscopy (Figs. 3 and 4). BHK-21 cell nucleus was stained with Hoechst dye and imaged under DAPI channel (Fig. 3A1–E1). Cells exposed to polymer NPs were imaged under FITC channel (Fig. 3A2–E2). Uptake of PVC NPs into the cells was followed over time by imaging the NP-exposed cells at regular intervals (1 min, 5 min, 15 min, 30 min and 1 h, Fig. 3A–E). After 15 min (Fig. 3,C3), many green dots were observed inside the cytoplasm corresponding to the presence of perylene dye encapsulated polymer NPs.

In the same way for PMMA internalisation, BHK-21 cells were stained with Hoechst and imaged under DAPI channel (Fig. 4A1–E1), NPs exposed cells were imaged under FITC channel (Fig. 4A2–E3) and both images were merged (Fig. 4A3–E3). Strong green signals were observed from the NPs exposed cells, which is consistent with the results reported earlier^28^. From the data, it appears that PMMA NPs enter the cells more readily than PVC NPs, considering the same size range for both particles. No background signal was observed in the absence of PMMA and PVC NPs (Figure S6). In addition, the cells exposed to the polymer NPs showed no distinct morphological changes, as compared with the untreated cells imaged under the same conditions (Figs. 3, 4).

### Tracking of nanoparticles inside BHK-21 cells

**Figure 5:**
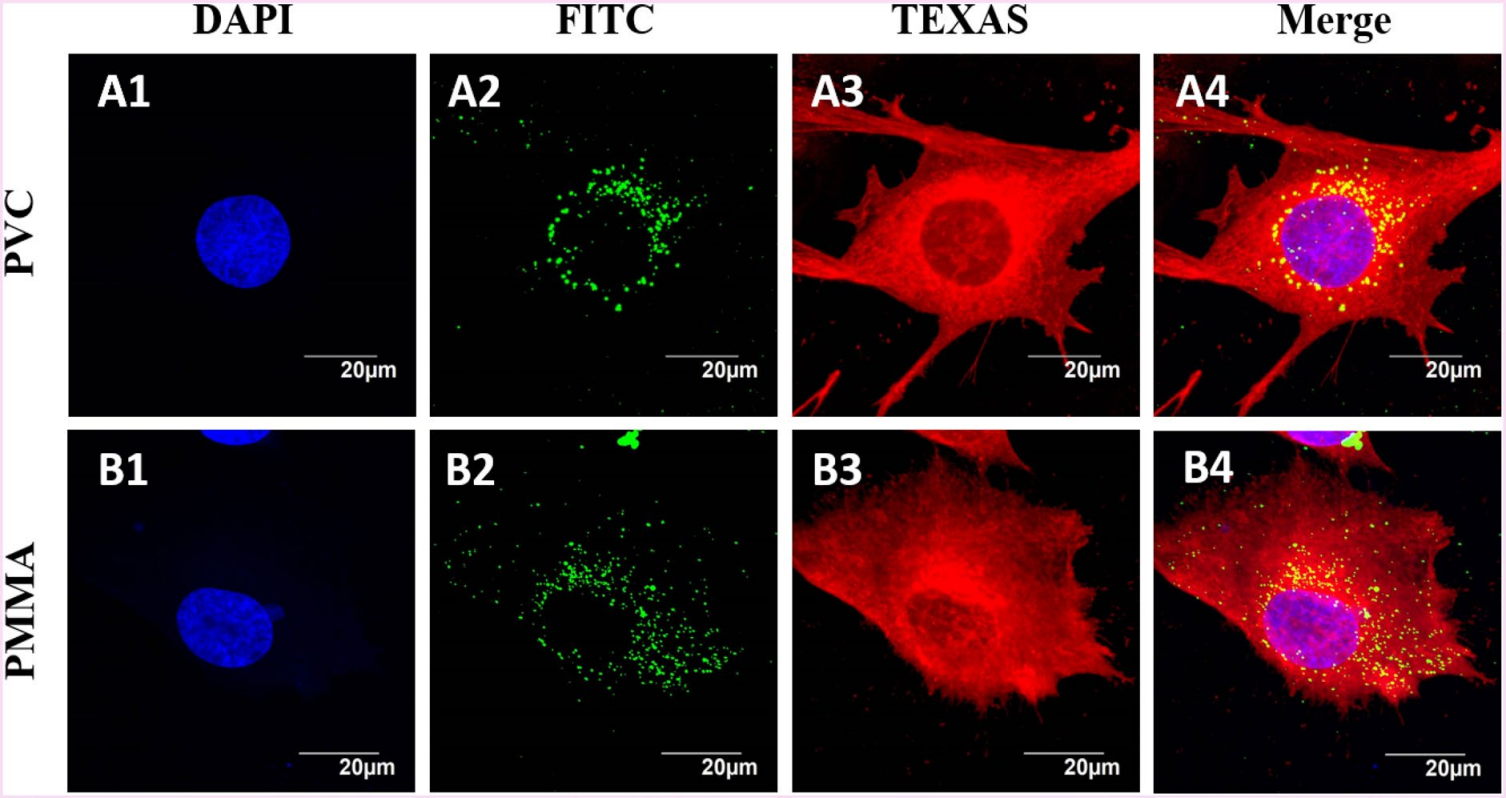
Confocal images of BHK-21 cells exposed to PVC (**A1** to **A4**) and PMMA (**B1** to **B4**) NPs at 200 µg/mL in RPMI media. Cell membrane was stained with cell mask deep red (invitrogen) and nucleus stained blue with Hoechst dye.

To assess the amount of polymer NPs internalised into BHK-21 cells, cell membrane was stained with Cell Mask (Cell mask Deep Red, Invitrogen) and Hoechst blue stain was used to stain nucleus (Fig. 5A1, B1). The intercellular uptake of polymer NPs after 24 h incubation was observed in the XY-plane and further examined by taking a set of cross sectional images in the Z-axis through the cell. Images of the cells exposed to green fluorescent polymer NPs were imaged under FITC green channel (Fig. 5A2, PVC and B2, PMMA). The cells were also imaged after treatment with TEXAS Red mask (Fig. 5A3–B3) to understand the changes in cell morphologies. After overlaying of images (Fig. 5A1–A3) or (Fig. 5B1–B3), shows more polymer NPs accumulated around the nucleus with no significant changes in cell morphologies (Fig. 5A4, B4). This is consistent with earlier reported results from HeLa cells exposed to medium sized (100–200 nm) PMMA nanoparticles showed a similar distribution pattern inside the cells^31^. Both PVC and PMMA NPs showed similar trends of internalization, which is also observed earlier for the cellular uptake in HeLa and T98G brain cell lines^7,32,33^.

### Colocalization experiments of plastic nanoparticles with Latex Beads

**Figure 6:**
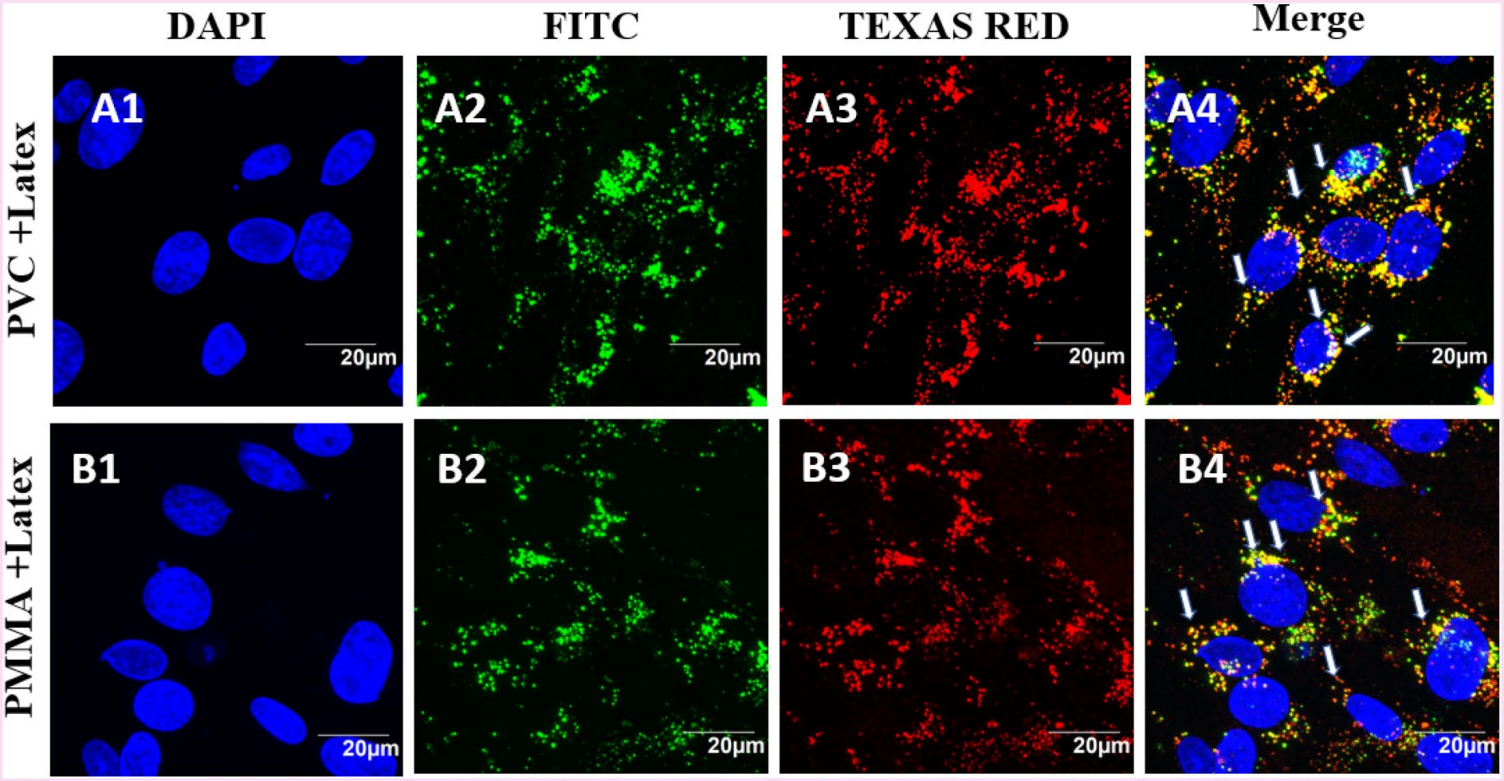
BHK-21 cells exposed to PVC (**A1**–**A4**) and PMMA (**B1**–**B4**) NPs at a concentration of 200 µg/mL for 24 h and latex beads (80 µg/mL). The images represent nucleus stained with Hoechst dye and cells exposed to PVC or PMMA NPs (green emitting, **A2**, **B2**) and red fluorescent latex beads (**A3**, **B3**). The blue, green and red fluorescent images are overlaid to show the localization of PVC and PMMA NPs and latex beads inside the cell, indicating that PVC and PMMA NPs are entering the cell by endocytosis.

Confocal microscopic imaging of the cells exposed to luminescent particles allows direct observation of binding, uptake and co-localization of NPs by the cells. To confirm that NPs are taken up into the endosomes, we sought to label the cells with an endosomal marker. Unfortunately, many commercially available antibodies against endosomal markers do not stain BHK-21 cells. Previous studies have demonstrated that latex beads of size less than 200 nm were internalized into the cells via endocytosis^34^. BHK cells were exposed simultaneously to a mixture of PVC or PMMA NPs and commercial latex beads (8 µL, 80 µM), and incubated for 24 h. The BHK-21 cell nucleus was stained and imaged under DAPI channel (Fig. 6A1, B1). The cells exposed to polymer (PVC and PMMA) nanoparticles were imaged under FITC green channel (Fig. 6A2, B2). Similarly, the cells exposed to latex beads and stained with TEXAS Red were imaged (Fig. 6A3, B3) under the same experimental conditions. The yellow emission from overlapping the red and green fluorescence indicated by arrows in Fig. 6A4, B4, clearly indicates that our polymer NPs and latex beads were colocalized inside the cells. It is understood that the polymer NPs are taken up by the cells via endocytosis, similar to the reported mechanism for the intake of latex particles^35^. No signal interference was observed in the presence of NPs and latex beads imaged under FITC and TEXAS red channels, respectively (Figure S7).

**Figure 7:**
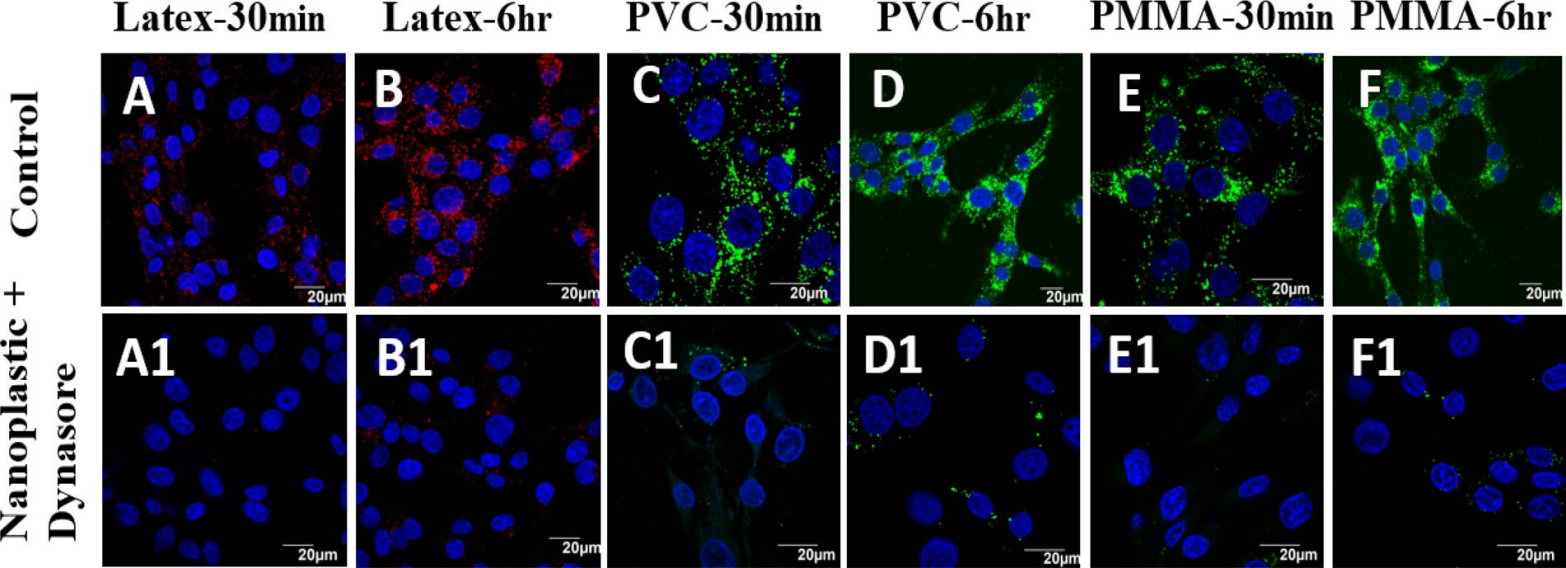
BHK-21 cells exposed to latex beads (**A**,**B**); PVC (**C**,**D**) and PMMA (**E**,**F**) NPs at a concentration of 200 µg/mL (**A**–**F**) and exposed to dynasore inhibitor (80 μM) and polymer NPs (**A1**–**F1**) at different time points. All images were taken under identical conditions for comparison. The images represent nucleus stained with Hoechst dye (blue), cells exposed to green fluorescent PVC or PMMA NPs and red fluorescence represent exposure to latex beads.

Cellular inhibitor was employed to determine the endocytosis pathway responsible for uptake and transport of nanoplastic particles. Dynasore is a non-competitive inhibitor of dynamin-dependent endocytosis in cells^36,37^. BHK cells exposed to Dynasore inhibitor and polymer NPs showed no particles inside the cells, implying the effective blockage of polymer particle transport into the cell during the first 6 h (Fig. 7A1–F1), which was confirmed with the control images taken without adding dynasore inhibitor (Fig. 7A–F). In the absence of dynasore inhibitor, the polymer NPs enter the cells within a short period (e.g. 30 min).

### Polymer NPs induced changes in cellular ATP content

**Figure 8:**
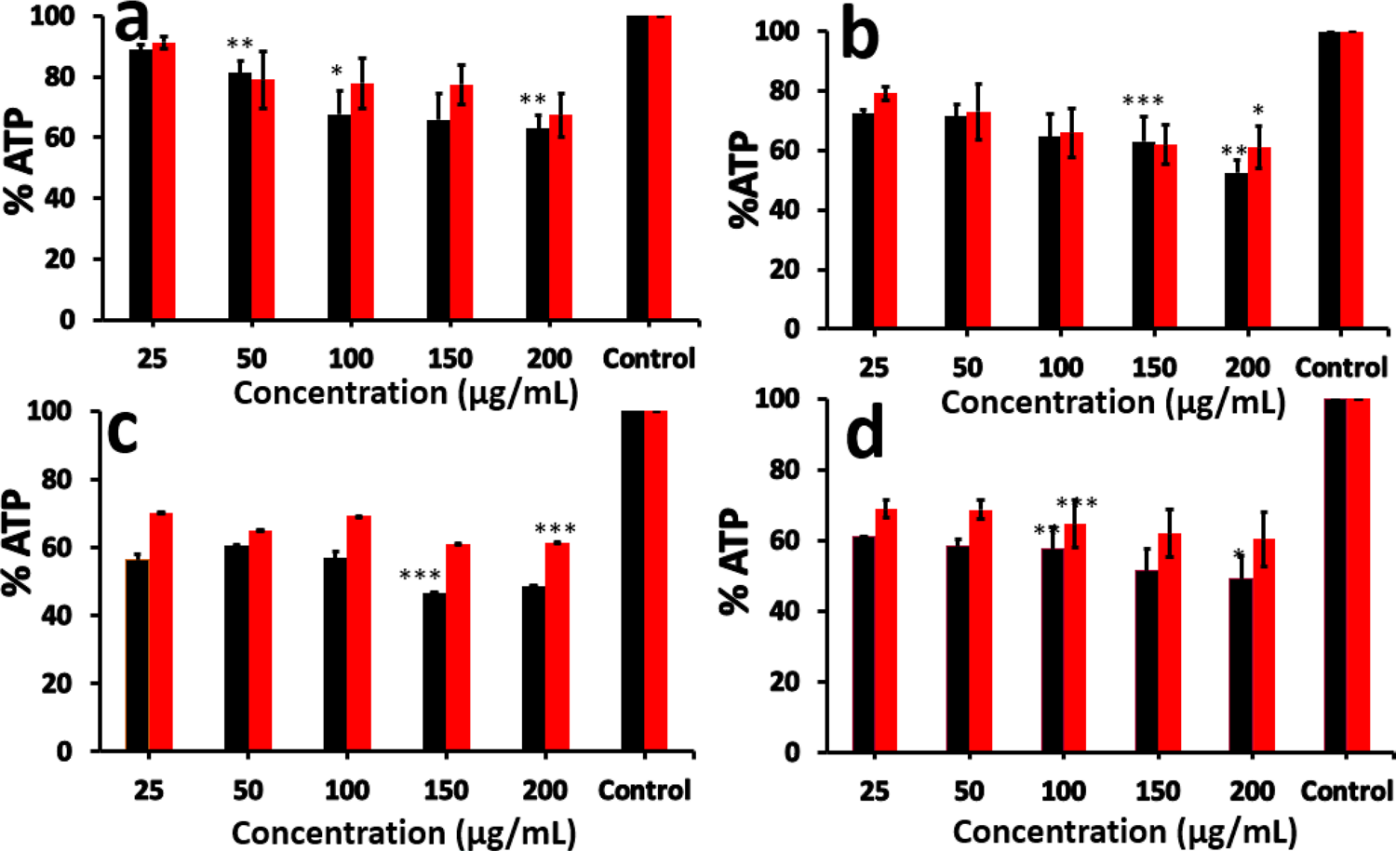
Intracellular concentration of ATP after exposure to PVC (■) and PMMA ( ) NPs at different concentrations for (**a**) 24 h, (**b**) 48 h, (**c**) 96 h and (**d**) 120 h. The data for 72 h incubation is given in the supporting information, Figure S8. The results are given as % ATP level with respect to control (untreated cells) and are represented as the mean ± SEM of three experiments. Statistical analysis was done by one-way ANOVA (*p < 0.05, **p < 0.01, ***p < 0.001, ****p < 0.0001) compared to control (untreated cells).

Adenosine triphosphate (ATP) is the energy currency of the cells and ATP content is a reflection of the cellular health in terms of energy production. ATP assays showed a time dependent drop in fluorescence intensity of oxyluciferin along with increase in concentrations of polymer NPs. The ATP content was decreased with increase in time and concertation of the polymer NPs (Fig. 8a–d). At 24 h, BHK-21 cells showed significant changes in the ATP production in presence of PVC (63.0 ± 4.4%) and PMMA (67.3 ± 2.7%) (Fig. 8a) nanoparticles at a concentration of 200 µg/mL. ATP content was decreased to 52.4 ± 4.4% in presence of PVC NPs and 61.0 ± 7.3% for PMMA NPs after 48 h (Fig. 8b). Similar decreasing trend was observed with increase in time to 72 h (Figure S8), 96 h (Fig. 8c) and 120 h (Fig. 8d). The results suggest that polymer NPs influence cellular energetics and ATP production.

### Analysis of reactive oxygen species (ROS)

The reactive oxygen species (ROS) such as superoxide anions (O_2_^−^) and hydroxide radicals are formed in the living system through redox processes inside the cells. ROS can be generated inside the cell via endogenous processes or interaction with toxic chemicals or nanomaterials^38,39^. In the present study, an increase in ROS production in presence of PVC (52.7%) and PMMA (25.4%) particles was observed inside the exposed cells.

**Figure 9:**
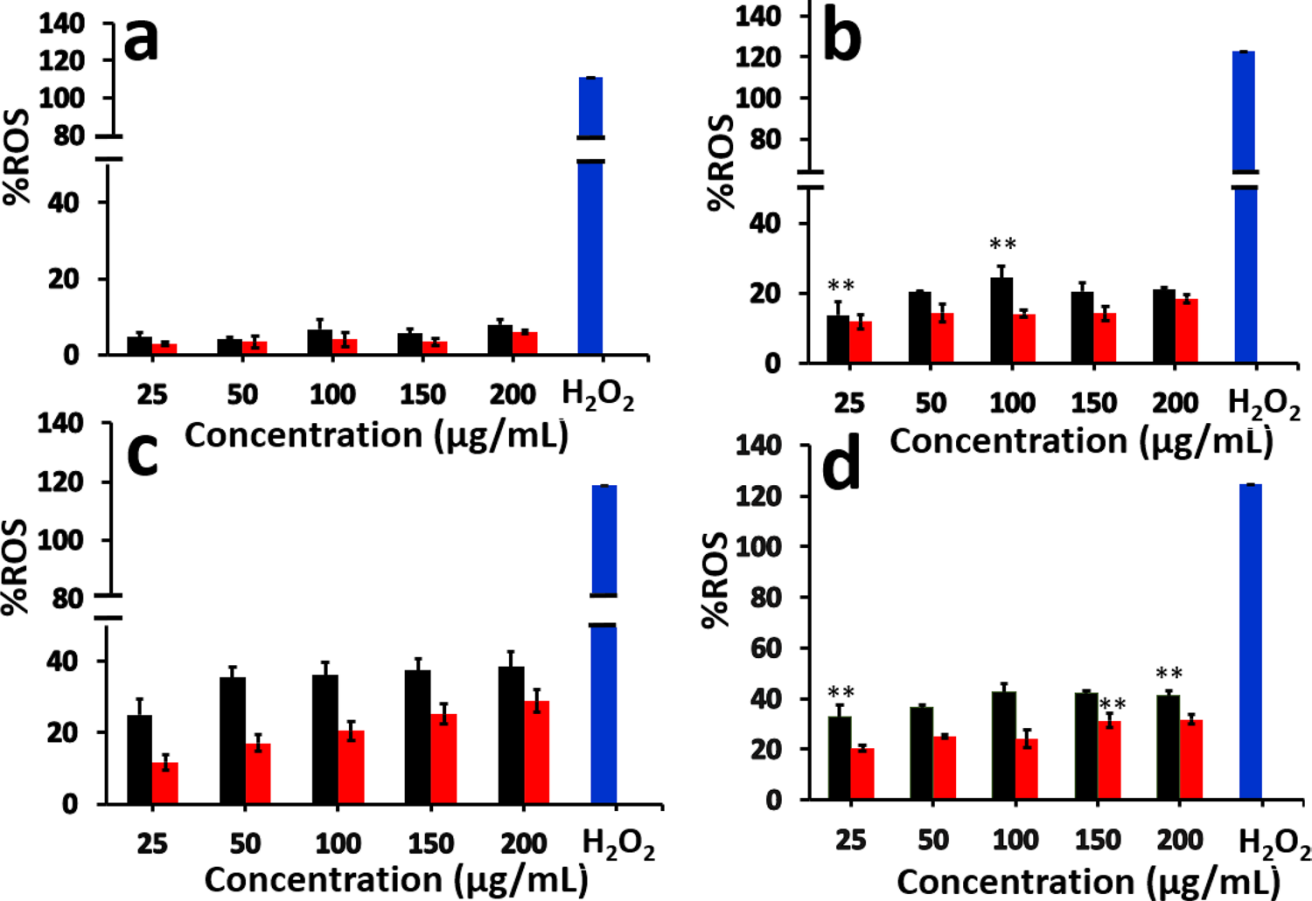
Concentration of ROS produced in BHK-21 cells after (**a**) 24 h, (**b**) 48 h, (**c**) 96 h and (**d**) 120 h of incubation with PVC (■) and PMMA ( ) NPs at different concentrations. The data for 72 h incubation were given in the supporting information, Figure S9. The results are given as % of the ROS production with respect to the control (untreated cells) and are the mean ± SEM of three experiments. Statistical analysis was done by one-way ANOVA (*p < 0.05, **p < 0.01, ***p < 0.001, ****p < 0.0001) compared to control cells with no particles added.

As shown in Fig. 9a, BHK-21 cells exposed to PVC and PMMA particles did not show significant changes in the ROS production after 24 h. At 24 h, the ROS production in presence of PVC (1.4%) and PMMA (0.5%) particles is rather modest but statistically significant as compared to control cells with no polymer NPs were added. But after 48 h (Fig. 9b), significant changes in cellular ROS concentrations were observed. Similarly, ROS concentration was increased at 72 h (Figure S9) and 96 h (Fig. 9c). After 120 h, the ROS concentration inside the cells exposed to PVC or PMMA NPs was increased to 52.7% and 25.4%, respectively, as compared to the control samples (Fig. 9d). For a positive control, the similar number of BHK cells in a separate well were treated with 10 µL of 0.1% H_2_O_2_ and incubated for 1 h, treated to DCF-DA solution and incubated for 1 h, before measuring the ROS concentration.

### Lactate dehydrogenase (LDH) assay

The effect of PVC and PMMA nanoparticles on the cell membrane integrity was studied by lactate dehydrogenase (LDH) assay. LDH is a large cytosolic enzyme present in mammalian cells that could leak out of the cells through the damaged cell membrane during necrotic cell death^40–44^. LDH assay is widely used in toxicology studies^45,46^ and rely on the measurement of LDH activity in the extra cellular medium. LDH catalyse the oxidation of lactate with concomitant reduction of NAD^+^ to NADH. Reductase uses NADH and reductase substrate to generate luciferin, which is converted to luminescent signal by luciferase enzyme.

**Figure 10:**
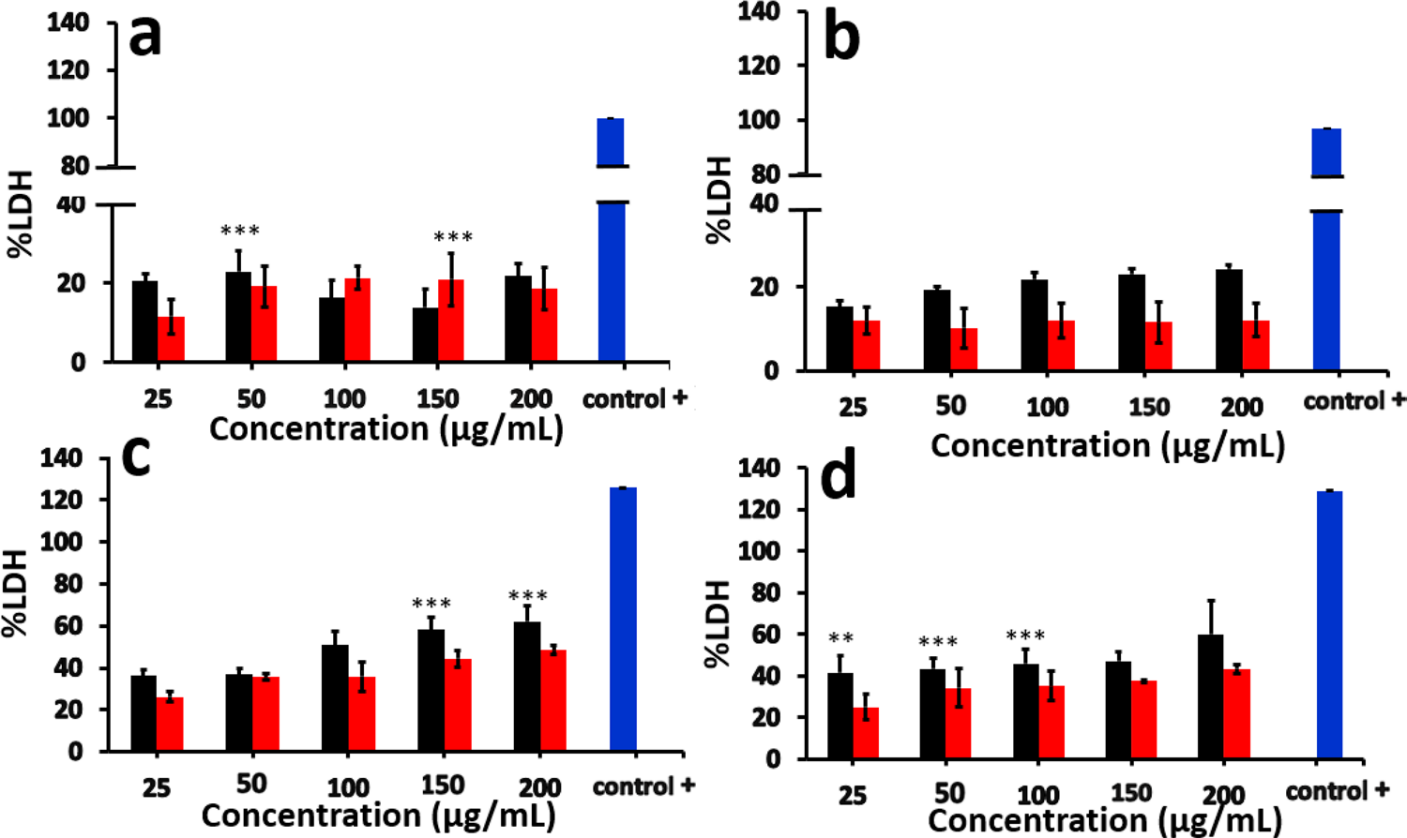
Concentration of LDH released into the medium after (a) 24 h, (b) 48 h, (c) 96 h, (d) 120 h, of incubation with PVC (■) and PMMA ( ) NPs. The data for 72 h incubation is given in the supporting information, Figure S10. The values represent as % LDH release with respect to control (untreated cells) and are the mean ± SEM of three experiments. Statistical analyses were done by one-way ANOVA (*p < 0.05, **p < 0.01, ***p < 0.001, ****p < 0.0001) compared to control. Unexposed cell lysis solution was used as positive control to obtain the maximum LDH release.

The amount of signal observed is directly proportional to the amount of LDH released to the medium after exposure of cells to polymer nanoparticles. In our experiments, an increase in LDH release in presence of PVC (21.7%) and PMMA NPs after 24 h (18.6%) (Fig. 10a) was observed. The amount of LDH was continued to increase with time (e.g. 48 h and 96 h, Fig. 10b,c) and reached to 60.2 ± 15.7% after 120 h (Fig. 10d) for PVC. For PMMA nanoparticles, similar trend was observed with the LDH concentration at 120 h was about 43.3 ± 2.3% (Fig. 10d). In presence of both PVC and PMMA NPs, LDH was detected after 24 h and reached maximum at 120 h. The data collected after 72 h are given in the supporting information (Figure S10).

### Cell cycle analysis

**Figure 11:**
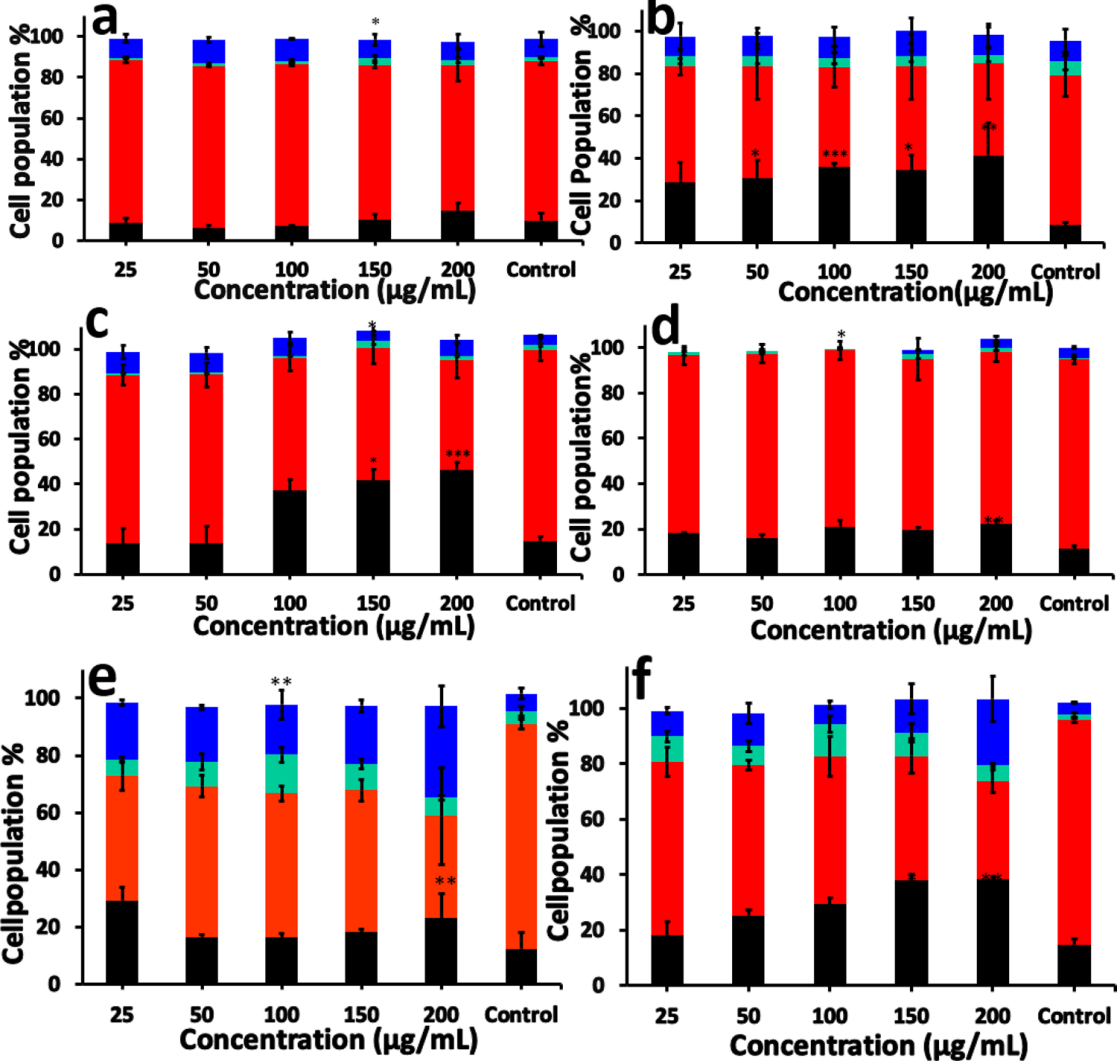
Cell cycle analysis after incubation with PVC NPs for (**a**) 24 h, (**b**) 48 h, (**c**) 72 h and PMMA NPs for (**d**) 24 h, (**e**) 48 h, (**f**) 72 h. SubG1 (■), G0/G1 ( ), S ( ), G2/M ( ). The results are given as % cell cycle distribution with respect to concentration. The values represent the mean ± SEM of three experiments. Statistical analysis was done by one-way ANOVA (*p < 0.05, **p < 0.01, ***p < 0.001, ****p < 0.0001) compared to the control.

Flow cytometric analysis of the BHK cells exposed to polymer NPs helps to visualize the cell population at different stages of cell cycle. We analysed the effect of PVC and PMMA nanoparticles on BHK-21 cell cycle distribution, which showed significant redistribution at different phases (Fig. 11). The PMMA and PVC NPs exposed cells showed the cell cycle arrest in a dose and time dependent manner (Fig. 11). The cell cycle pattern was not affected by nanoparticles (PVC and PMMA) until 24 h (Fig. 11a,d), but shows significant changes after 48 h (Fig. 11b,e). After exposing the cells to the polymer NPs for 72 h, more number of cells were accumulated under SubG1 phase for PVC exposed cells than PMMA NPs exposed ones (Fig. 11c,f). Exposing cells to PVC NPs for 72 h showed that 46.5% ± 3.1 cells were accumulated at Sub G1 phase, 48.7 ± 7.8% cells at Go/G1 phase and 7.3 ± 1.7% cells at G2/M phase. Similarly, cells exposed to PMMA nanoparticles, 38.3 ± 0.8% cells are accumulated at sub G1 phase, 48.7 ± 7.8 cells at G0/G1 phase and 23.9% ± 8.2 cells accumulate at the G2/M phase after 72 h, which indicates that the cells are undergoing apoptosis.

## Discussion

Recently, a few studies have been focused on understanding the impact of polymer micro- or nanoparticles (MPs/NPs) on human cells. However, there are significant gaps in existing data to clearly identify the toxicological effects of different polymer nanoparticles on a single cell line. Here, we explore the impact of the exposure of nanoparticles prepared from two common polymers, PVC and PMMA, on BHK-21 cell lines.

Previous studies have reported that microplastic particles induce oxidative stress and reduction in cell viability in HeLa cells^44^. At high concentration, PVC NPs showed toxicity to BHK-21 cells in terms of low cell viability 40.3 ± 0.1% after 120 h, whereas PMMA particles showed 61.3 ± 4.0% viability under the same conditions. PVC and PMMA NPs induce toxicity in a dose dependent manner (Fig. 2). Control experiments were done to demonstrate that the observed toxicity is due to polymer NPs and not caused by the presence of unreacted substances or solvents in the solution (Figure S2, S3 and S4). Using a time dependant study, we have shown that the polymer NPs are taken up by the cells within a few minutes of exposure (Figs. 3, 4). Since both particles have similar surface charges (Table 1), small differences in uptake of PVC and PMMA NPs could be due to the differences in size. PVC NPs are smaller in size (120 nm) as compared to the PMMA NPs (140 nm, Table 1). Further investigations are need to establish the reasons behind the observed differences in intake of the two polymer NPs.

The mechanism of NP uptake by BHK-21 cells was investigated using confocal microscopy. After incubation of BHK-21 cells with polymer particles, it can be seen that both polymer NPs enter the cells readily and distributed inside the cytoplasm. So far, no detectable concentration of particles is seen inside the nucleus. In addition, the cellular morphologies are not affected by the exposure of BHK-21 cells to polymer NPs (Fig. 5). The confocal images show that the particles do enter the cells and gets accumulated inside the cytoplasm. A few studies using TiO_2_ and peptide conjugated Au NPs showed accumulation of such particles in the perinuclear region of the mouse neural stem cells and malignant epithelial cell membrane, respectively^47,48^. Similar observations were also reported from Caco-2 cells exposed to PMMA nanoparticles and Hela cells exposed to PS NPs^31,49^. After 24 h, more number of PVC and PMMA NPs were accumulated at the perinuclear region of the BHk-21 cells. Our results from exposing BHK-21 cells to PVC and PMMA NPs are consistent with earlier literature reports.

Cellular uptake of NPs is usually occurring via three different types of mechanisms, which involves endocytosis, phagocytosis and pinocytosis. Since the cell uses phagocytosis to engulf large particles or even the whole cell and pinocytosis for small particles through fluid assisted intake of solutes, we presume that the observed entry of our medium sized polymer NPs could be due to endocytosis^37^. To confirm this mechanism of internalisation of particles, an already established method of colocalization of latex beads with polymer NPs was used. Latex beads are known to enter the cells via endocytosis^50,51^. Both PVC and PMMA NPs are colocalised with latex beads (size < 200 nm) within 24 h of treatment, suggesting that the polymer nanoparticles are able to internalise into the cells via dynamin dependent endocytosis (Fig. 6)^36,41^. From the Dynasore inhibition study (Fig. 7) carried out to further confirm the mechanism, the uptake of polymer NPs is blocked by the Dynasore inhibitor, confirming the endocytosis pathway for the uptake of polymer NPs. As evident from A1–F1 (Fig. 7), the Dynasore treated cells did not show red or green fluorescence in a significant manner, indicating the lack of uptake of the polymer NPs by the cell. The impacts of exposure of polymer NPs to the BHK cells were further analysed using ATP, ROS and LDH assays. The polymer NPs exposure led to a time and dose dependant decrease in ATP production (52% for PVC and 61% for PMMA) in BHK cell lines. This is consistent with the observed cytotoxicity and energy assisted endocytosis mediated uptake of the particles into the cells. Such a decrease in ATP concentration (Fig. 8) indicates the cell activities are perturbed by the presence of polymer NPs. Similarly, exposure to PVC and PMMA NPs led to a time and dose dependant increase in concentration of ROS (52% for PVC and 25% for PMMA) in BHK-21 cells (Fig. 9). The effect of membrane integrity of BHK cells was also studied using LDH assay (Fig. 10) in presence of both NPs. After 120 h, the LDH release was increased and reached 62% for PVC and 48% for PMMA NPs (Fig. 10). This implies some damage to the cell membrane after exposure to the polymer particles. The cell cycle process involves duplication of cellular DNA, followed by the division of cytoplasm and organelles to produces two daughter cells^52^. Cell cycle progression has five known phases, which includes G0 (gap 0), G1 (gap1), S (DNA synthesis), G2 (gap 2) and M (mitosis). In between these phases, two important check points are at the G1/S and G2/M boundaries. PVC and PMMA NPs exposed cells showed an accumulation in G0/G1 phase, sub G1 and G2/M phases after 72 h which indicate that the cells are undergoing apoptosis (Fig. 11). The accumulation of cells at G0/G1 phase implies that the cells are not able to get sufficient nutrients for forward progression and represent a state of quiescence. The cells that gets arrested in sub G1 phase indicate DNA damage which could initiate apoptosis. Similarly, the cells with DNA damage could also get arrested in G2/M phase and do not undergo mitosis.

Among the PVC and PMMA NPs studied, the data shows that PVC NPs induces higher ROS production and lower the ATP concentration, which causes higher cell death via apoptotic pathway. Both PVC and PMMA samples were purchased as powders from commercial sources. The observed differences in cellular activities between the PVC and PMMA NPs could be due to a few factors such as inherent chemical structure of the polymers, nature of the stabilizers added by the manufacturer, distribution in molecular weight and traces of catalysts left over during polymerization. For example, PVC contains a significant number of C–Cl bonds, which are relatively easy to cleave under oxidizing conditions leading to the formation of chloride radicals, which are toxic to cells. Other factors are much more complicated and need more time to investigate and establish their contribution to the observed differences in toxicity or cellular response. PVC is also more hydrophobic in character than PMMA polymer. Such factors could be the main reason for PVC is not used in many biomedical applications. Both PMMA and hydrolysed poly(methacrylic acid) polymers are nontoxic to living cells and used in biomedical applications. We are currently analysing the commercial samples to identify the mechanism and will report the details in the near future. The current study completed under identical conditions using BHK cells highlights the comparison of activities of PVC and PMMA nanoparticles. For this work, relatively high concentrations (25, 50, 100, 150, 200 µg/mL) of particles were selected for toxicity evaluation. This is due to two reasons, which include no clear understanding of the exact concentration of micro- or nanoplastic particles in the environment and higher concentrations of the nanoplastics are expected to induce biological response inside the cells faster than low concentrations. More studies involving multiple cell lines are needed to understand the full impact of plastic NPs in humans.

In this study, we explored the impact of PVC and PMMA NPs on BHK-21 cell lines at different concentrations and exposure time. Luminescent PTE encapsulated PVC and PMMA nanoparticles were synthesized by rapid nanoprecipitation technique and fully characterised. The physio-chemical properties of the nanoplastic particles were studied by different characterization techniques. The fluorescent plastic NPs were internalized into the cellular cytoplasm, but did not enter the nucleus. The exact reason for this could be due to both structural and functional differences in both cell membrane and nuclear membrane. Both colocalization experiments and the dynasore inhibition studies showed that endocytosis is the predominant pathways for the uptake of PVC and PMMA NPs by the BHK cells. Moreover, significant changes (40% for PVC and 61% for PMMA) in the cell viability was observed for BHK cells after exposure to polymer NPs. Similarly, the exposure to polymer NPs caused a decrease in ATP production, increase in ROS concentration and significant LDH release from BHK cells. More studies are required to determine other factors associated with uptake of PVC and PMMA NPs and results will be published in our upcoming publications.

## Materials and methods

All high purity chemicals, PMMA (Mw-15000), PVC (Mw-120000), Latex beads, sodium dodecyl sulphate (SDS), acetone, tetrahydrofuran (THF) were purchased from Sigma Aldrich and used without further purification. Deionised sterilised water was used for all experiments. Baby Hamster Normal Kidney Fibroblast cells (BHK-21) were purchased from American Type Culture Collection (ATCC, USA). The cells were cultured in RPMI 1640 medium (Biowest, France), which was supplemented with 10% fetal bovine serum (FBS) and 1% of 100X Penicillin–Streptomycin (Thermo Fisher Scientific, Waltham, USA). Cells incubated at 95% humidity, 37 °C in 5% CO_2_ atmosphere. The cells were enzymatically detached using trypsin-ethylenediamine tetra acetic acid (Trypsin–EDTA) solution and subcultured in a new T-25 flask and used for all experiments. DCFDA assay and Alamar blue assay kits were purchased from Invitrogen (Thermofisher Scientific Waltham, USA). LDH assay and ATP assay kits were purchased from Promega Pvt Ltd and Abcam, respectively. The cell mask red was purchased from Invitrogen (Thermo fisher scientific, Waltham, USA).

### Instrumentation

UV–Vsible spectra were measured using a UV-1800 Shimadzu UV–VIS spectrophotometer. Emission spectra were recorded on an Agilent Cary Eclipse Fluorescence Spectrophotometer using an excitation wavelength of 550 nm corresponding to the absorption maximum of the perylene dye. Scanning electron micrographs (SEM) were recorded using a JEOL JSM-6701F Field emission scanning electron microscope (FE-SEM). All samples were prepared by diluting the stock solution of polymer NPs with sterilised water to a concentration between 0.05 and 0.75 mg/mL. For SEM imaging purpose, the samples were drop casted on glass cover slips, dried and were coated with platinum before imaging. Dynamic light scattering (DLS) and zeta potentials were measured using a Malvern Zeta Size instrument. Measurements were carried out at 25 °C, with refractive index of polymers set at 1.489. The average value of three sets of data were taken as the particle size of the prepared polymer particles. All images were recorded using an Olympus FV1000 confocal microscope.

### Nanoprecipitation of particles

Nanoprecipitation is a kinetically controlled process where parameters such as concentration of the solution, temperature, addition rate and presence of stabilizer influence the formation of monodispersed and stable particles^24–28^. Polymer NP dispersions were synthesised according to a published procedure^28^ in which polymer (400 mg), SDS (10 mg, 4 wt% of polymer), and the perylene tetrabutylester dye (10 mg, 4 wt% of polymer) were dissolved in acetone or tetrahydrofuran (50 mL). The clear solution (5 mL) of the mixture was poured immediately into 50 mL of sterilised water, stirred overnight for the slow evaporation of the organic solvent and filtered using a cotton plug to remove large particles and impurities. The filtrate was dialysed using spectra/Por dialysis membrane MWCO1000. The resulted solution of the fluorescent polymer NPs was characterised by using dynamic light scattering (DLS), scanning electron microscopy, UV–Vis absorbance and fluorescence spectroscopy.

### Cell line and culture conditions

Baby Hamster Normal Kidney Fibroblast (BHK-21) cells were selected for exploring the uptake and cytotoxicity investigations of PVC and PMMA NPs. Experiments were conducted using RPMI 1640 cell culture medium supplemented with 10% FBS (Foetal Bovine Serum) and 1% of Antibiotic–Antimycotic 100X (Biowest) antimicrobial compound. The cells were cultured at 37 °C in a humidified atmosphere with 5% CO_2_ gas. After achieving the desired confluence, cells were enzymatically detached with trypsin—ethylenediamine tetra acetic acid (Trypsin–EDTA) and subcultured in a new T75 flask.

### Tracking of nanoparticles inside the cell

In order to follow the translocation of plastic nanoparticles, BHK-21 cells were exposed to fluorescent PMMA and PVC NP solutions at different concentrations. BHK-21 cells (10,000 cells/ well) seeded on a cover slip and placed in a well of a 24 well plate and incubated for 24 h at 37 °C and 5% CO_2_ atmosphere. After 24 h, the cells were exposed to PMMA or PVC NP solution (200 µg/mL) and incubated for 0, 1, 5, 15, 30 and 60 min. The cells were washed with PBS, fixed with 4% paraformaldehyde for 20 min at 37 °C, washed with PBS, stained with Hoechst blue dye solution and washed again with PBS to remove the excess reagents. The washed cover slip was mounted on a glass slide and imaged using a confocal microscope. To understand the particle internalization, cells were stained with cell mask (0.25 µg/mL, Deep Red Cell Mask, Invitrogen) in PBS for 10 min. The internalization of plastic NPs was visualized using a Olympus FV1000 confocal microscope with a resolution of 1024 × 1024 pixels. All experiments were carried out in triplicates.

### Colocalization of plastic nanoparticles with latex beads

In order to understand the colocalization of PVC and PMMA NPs, BHK-21 cells were exposed with synthetic polymer nanoparticles and commercially obtained latex beads. BHK—21 cells (10,000 cells/ well) were seeded on a cover slip in 24 well plate, incubated for 24 h and exposed to PVC, PMMA (200 µg/mL) nanoparticles for another 24 h at 37 °C in 5% CO_2_ atmosphere. Latex beads (80 µM, 100 nm) also incubated along with PVC and PMMA NPs for 24 h. The cells were washed with PBS, fixed with 4% paraformaldehyde for 20 min at 37 °C, washed 3 times with PBS, stained with Hoechst dye solution and washed again three times with PBS to remove the excess reagents. The cover slip was mounted on a glass slide and imaged using a confocal microscope. To check the signal interference, PVC NPs and Latex bead particles (10µL) were mixed, mounted on the coverslip and imaged under the same condition and considered as the control image (Figure S7).

To validate the endocytosis uptake pathways, 10,000 BHK-21 cells were seeded on a coverslip in a well plate and incubated for 24 h. BHK-21 cells exposed to a recommended dose of Dynasore (80 µM, Sigma Aldrich) for 30 min. The inhibitor was then removed, cells were washed with PBS and appropriate amount of PVC or PMMA NPs solution was added. In control experiments, polymer NPs (PVC or PMMA, 200 µg/mL) were added without the inhibitor. The cells were fixed in 4% paraformaldehyde for 40 min at room temperature, washed thrice with PBS, stained the nucleus with Hoechst dye solution and washed again three times with PBS to remove the excess reagents. The cells were imaged using a confocal microscope.

### Effect of SDS on cell viability

To understand the effect of SDS on cell viability, a solution of SDS in (50 mL, 4%) was prepared and appropriate volume of the solution (5 mL) was added rapidly into water (50 mL) to reach a final concentration of 0.4%, as similar to the final concentration in polymer NP solution. Another control was prepared at high concentration (4%) by dissolving SDS directly into water to study the effect of high concentration. An aliquot (10 µL) of each solution was added to BHK-21 (5000 cells/well) cells cultured inside a 96 well plate. After 24 h incubation at 37 °C in 5% CO_2_ atmosphere, the cell viability was checked by Alamar blue assay. The absorbance intensity was measured at 570 nm and 600 nm by using a microplate reader. Untreated cells were used as the control, and pure medium served as the background. Viability was determined with respect to the control (i.e. untreated cells).

### Effect of solvent interference cell viability

In order to understand the effect of solvent left over from the synthesis, appropriate amounts of pure acetone or tetrahydrofuran (THF, 5 mL) was added into water (50 mL) and left stirring for overnight to evaporate the organic solvent. An aliquot (10 µL) of the solution was added to BHK-21 cell (5000 cells/wells) seeded in 96-well plates. After 24 h of incubation at 37 °C and in 5% CO_2_ atmosphere, the cell viability was checked using Alamar blue assay. The absorbance intensities were measured at 570 nm and 600 nm using a microplate reader. Untreated cells were used as the control, and pure medium served as the background. Viability was determined with respect to the control (untreated cells).

### Cytotoxicity assay

The effect of polymer NPs on BHK-21 cells was studied by evaluating cell viability and measuring their metabolic activity using Alamar blue assay^28^ (Cell proliferation Assay from Promega). Briefly, BHK-21 cells were seeded in 96 well plate with 5000 cells / well. The cells were incubated for 24 h and exposed to PVC and PMMA NPs at different concentrations of 25, 50, 100, 150 and 200 µg/mL. After exposure, Alamar blue solution (10 µL) was added, incubated for 2 h, absorbance intensities at 570 nm and 600 nm were recorded using a microplate reader. Untreated cells were used as the control, and wells without cells were served as the background. Viability was determined with respect to the untreated cells.

### Adenosine triphosphate (ATP) assay

For the ATP assay, 5000 cells per well were plated and treated with different concentrations of polymer NPs (25, 50, 100, 150, 200 µg/mL) for a period of 24, 48, 72, 96 and 120 h. After incubation, the media was removed, and the cells were washed with PBS solution. Fresh PBS (80 µL) solution was added to the wells, followed by ATP assay reagent (20 µL). The plates were incubated for 1 h and the fluorescence was measured using an excitation wavelength of 485 nm and an emission wavelength of 600 nm. Cells with no plastic NPs were considered as controls. In this assay, ATP present in the living cells react with luciferin in the presence of luciferase enzyme and produce oxyluciferin which emits at 560 nm^39^. Thus, the observed fluorescent signal is proportional to the concentration of ATP present in the cells.

### Reactive oxygen species (ROS) assay

Intracellular ROS generation was monitored by employing 2,7-dichlorodihydrofluorescein diacetate (DCF-DA, Invitrogen) staining. DCF-DA shows an intense fluorescence, which is proportional to the concentration of ROS in the medium^53,54^. Dose and time dependent measurements of the ROS were done by incubating 5000 cells with polymer NPs at different concentrations (25, 50, 100, 150 and 200 µg/mL) for 24, 48, 72, 96, and 120 h followed by addition of DCF-DA solution (10µL, 5 µM) and incubated for 1 h at 37 °C in the dark. The plates were read under microplate reader at an excitation wavelength of 485 nm and emission wavelength of 535 nm. Cells exposed to hydrogen peroxide (0.1%, 10 µL) were used as a positive control.

### Lactate dehydrogenase (LDH) assay

Since, most cellular intake of plastic NPs occurs through endocytosis, the stability of the BHK-21 cell membrane was tested using the lactate dehydrogenase assay (LDH). The LDH - Glo cytotoxicity assay (containing lactate, NDH^+^, reductase, reductase substrate and Ultra-Glo rLuciferase) provides a simple bioluminescent method for quantifying LDH release. LDH catalyse the oxidation of lactate with concomitant reduction of NAD^+^ to NADH. Reductase uses NADH and reductase substrate to generate luciferin, which is converted to a bioluminescent signal by Ultra-Glo rluciferase^45,46^. The light signal generated is proportional to the amount of LDH present. Cells (5000 per well) were seeded in 96 well plate and incubated with nanoparticles different concentrations (25, 50, 100, 150, and 200 µg/mL) for 24, 48, 72, 96, and 120 h. Cell lysis solution was used as positive control to obtain the maximum LDH release. After different time of incubation, 50 µL of the media was removed without disturbing the cells at the bottom of the plate and transferred to another 96 well plate. Equal volume (50 µL) of LDH detection reagent solution was added to the well and incubated for 60 min at 37 °C. The fluorescence was measured under a microplate reader using an excitation wavelength of 560 nm and emission wavelength of 590 nm.

### Cell cycle analysis

In order to understand the effect of nanoplastic particles on cellular development, cell cycle analysis was performed by staining DNA with propidium iodide (PI) followed by cytometric measurement of fluorescence^55^. For cell cycle analysis, 1.2 × 10^6^ cells were seeded in 60 mm culture dish and incubated for 24 h at 37 °C in 50% CO_2_ atmosphere. After 24 h, cells were exposed to polymer NP solution at different concentrations (25, 50, 100, 150 and 200 µg/mL). A control well was setup with the same number of cells with no polymer NPs were added. The plates were incubated (24, 48, 72, 96 and 120 h), the media was collected from each well into separate falcon tubes (15 mL), the cells were washed with PBS and collected. The cells were tripsenized and centrifuged at 1500 rpm for 3 min. The pellet was washed with PBS, fixed in ice cold ethanol (70%) and stored at − 20 °C. Propidium Iodide staining solution was prepared by mixing 500 µL of propidium iodide and 50 µL of RNase solutions and diluted to 10 mL with PBS. After 1 h, the cells were washed again with PBS and stained for 60 min with propidium iodide staining solution (500 µL, 5 µg/mL). Samples were filtered and flow cytometry analysis was performed using BD LSR Fortessa cell analyser (BD Biosciences, San Diego, USA) at an excitation wavelength of 488 nm and emission wavelength of 610 nm.

## Supplementary Information

Supplementary Information
