# Supplementary material for: Understanding the interactions of poly(methyl methacrylate) and poly(vinyl chloride) nanoparticles with BHK-21 cell line
Source: Sci Rep. 2021 Jan 22;11:2089. doi: 10.1038/s41598-020-80708-0 (PMC7822812; doi:10.1038/s41598-020-80708-0)
Supplement: Supplementary file 1 — Supplementary Information [file 41598_2020_80708_MOESM1_ESM.docx]

**Understanding the interactions of poly(methyl methacrylate) and poly(vinyl chloride) nanoparticles with BHK-21 cell line**

Gomathi Mahadevan and Suresh Valiyaveettil ^*^

Department of Chemistry, National University of Singapore, 3 Science Drive 3, Singapore 117543. Email. chmsv@nus.edu.sg

**Supporting Information**

**Contents**

1. Physical Characterization S2

DLS data S2

1. Effect of SDS on BHK -21 cells S2
2. Effect of traces of organic solvent on BHK -21 cells S3
3. Effect of supernatant solution on BHk-21 cell Viability S3
4. Effect of dye encapsulated nanoparticles on BHK cell viability S4
5. Control experiments with BHK – 21 cells with no nanoparticles added. S5
6. Control experiment for background signal interferences S6
7. Effect of nanoparticles on different cellular processes S7

Effect of nanoparticles on intracellular ATP production S7

Effect of nanoparticles on intracellular ROS production S7

Effect of nanoparticles on intracellular LDH release S8

1. **Physical characterisation**


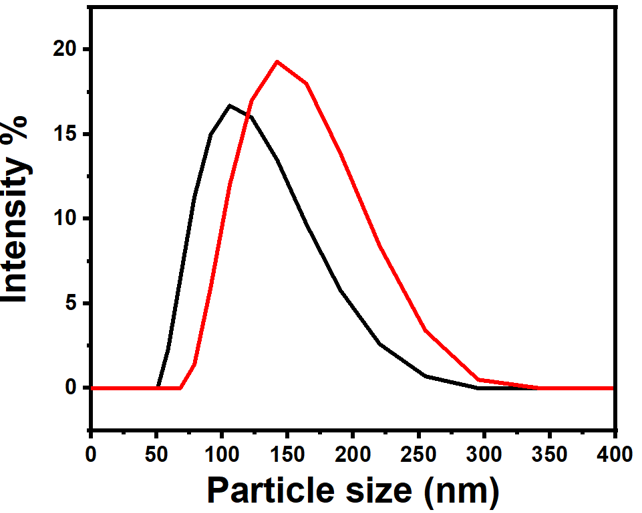


**Figure S1.** DLS data of polymer NPs prepared from nanoprecipitation and particle size distribution of (**—**) PVC and (**—**) PMMA NPs.

1. **Effect of SDS on BHK -21 cells**


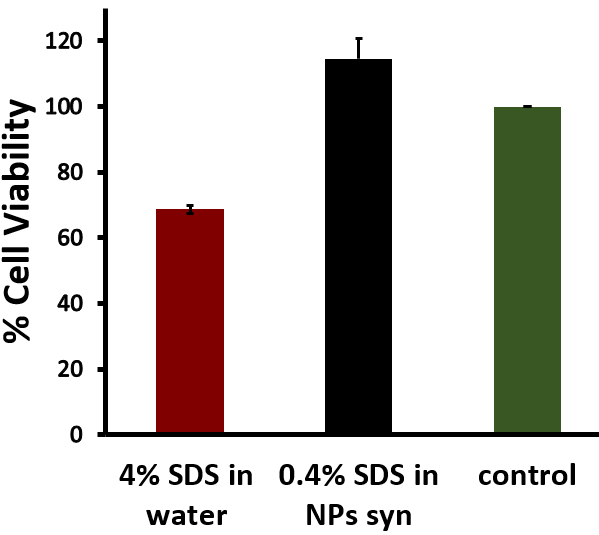


**Figure S2.** Effect SDS on cell viability. The results are given as % of the viability of the control and mean values of 3 independent experiments. Statistical analysis was done by one-way ANOVA (*p < 0.05, **p < 0.01, ***p < 0.001, ****p < 0.0001) and compared with control sample without polymer NPs added.

1. **Effect of traces of organic solvent on BHK -21 cells**


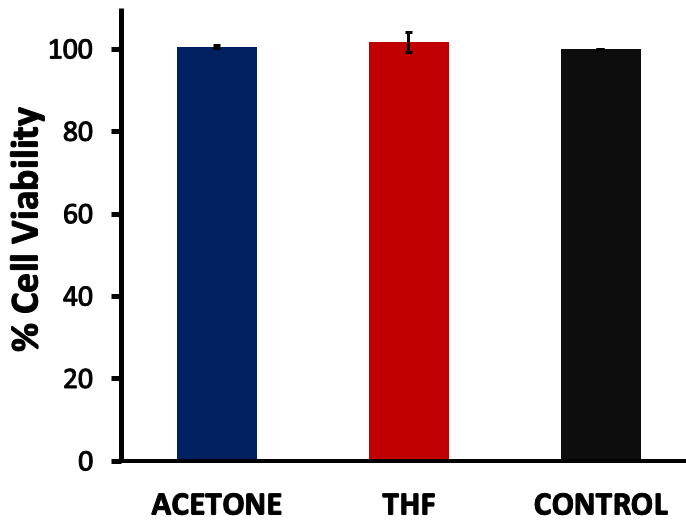


**Figure S3.** Cell viability of BHK cells exposed to trace amounts of acetone or tetrahydrofuran (THF). The results are given as % of the viability of the control and mean values of n = 3 independent experiments. Statistical analysis was done by one-way ANOVA (*p < 0.05, **p < 0.01, ***p < 0.001, ****p < 0.0001) and compared with control with no polymer NPs added.

**4**. **Effect of supernatant solution** **on BHK-21 cell viability**


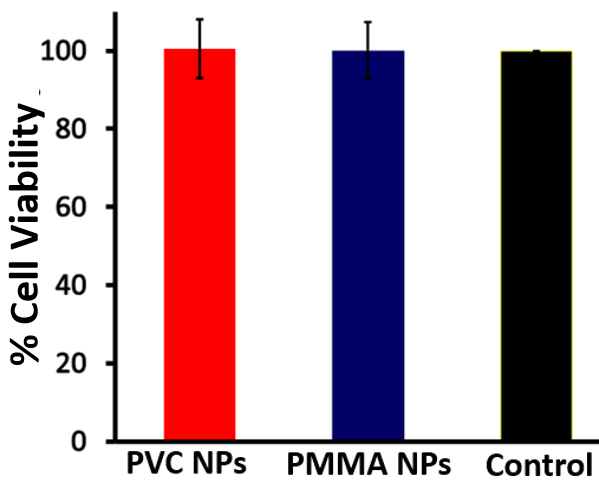


**Figure S4**. Effect of supernatant solution (which is expected to contain some SDS or trace amounts of organic solvents) on cell viability. The results are given as % of the viability of the control, mean ± SEM values of n = 3 independent experiments. Statistical analysis was done by one-way ANOVA (*p<0.05, **p<0.01, ***p<0.001, ****p<0.0001) and compared with control with no polymer NPs added.

1. **Effect of dye encapsulated NPs on BHK cell viability**


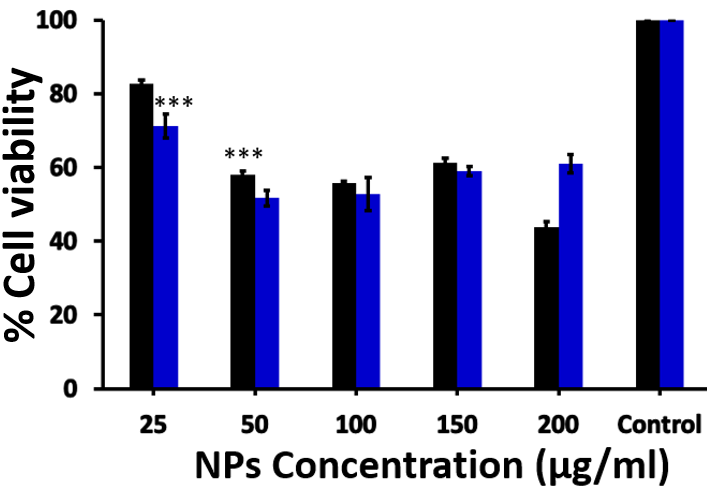


**Figure S5.** The viability of BHK-21 cells exposed to different concentrations of PVC (◼) and PMMA (◼) NPs and incubated for 72h. The results are given as % of the viability of the control and mean values of n = 3 independent experiments. Statistical analysis was done by one-way ANOVA (*p < 0.05, **p < 0.01, ***p < 0.001, ****p < 0.0001) and compared with control with no polymer NPs added.

1. **Control experiments with BHK – 21 cells with no polymer NPs added.**


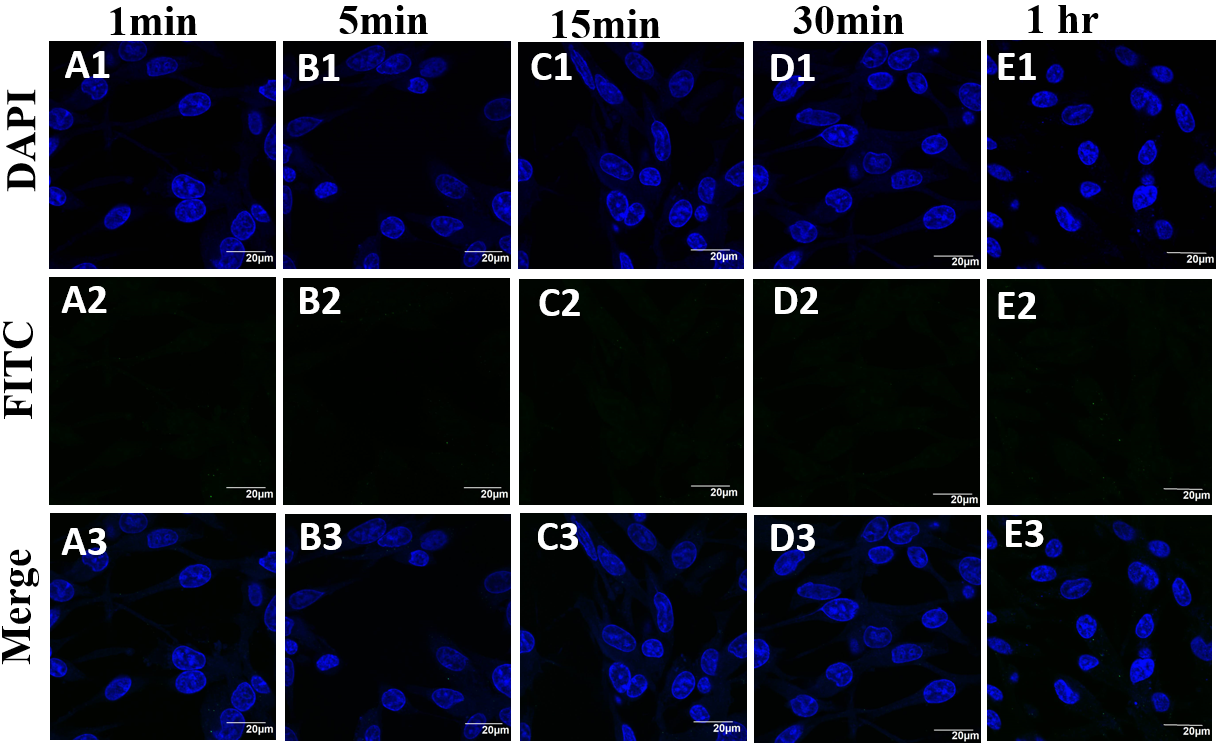


**Figure S6.** Confocal images of the BHK-21 cells as control (untreated with NPs) after 1 min (A1-A3), 5 min (B1-B3), 15 min (C1-C3), 30 min (D1-D3) and 1 hr (E1-E3). From top to bottom rows: DAPI stain in blue channel, FITC stain in green channel, overlay of blue and green channels. The scale bar = 20µm.

**7. Control experiment for the back ground signal inferences.**


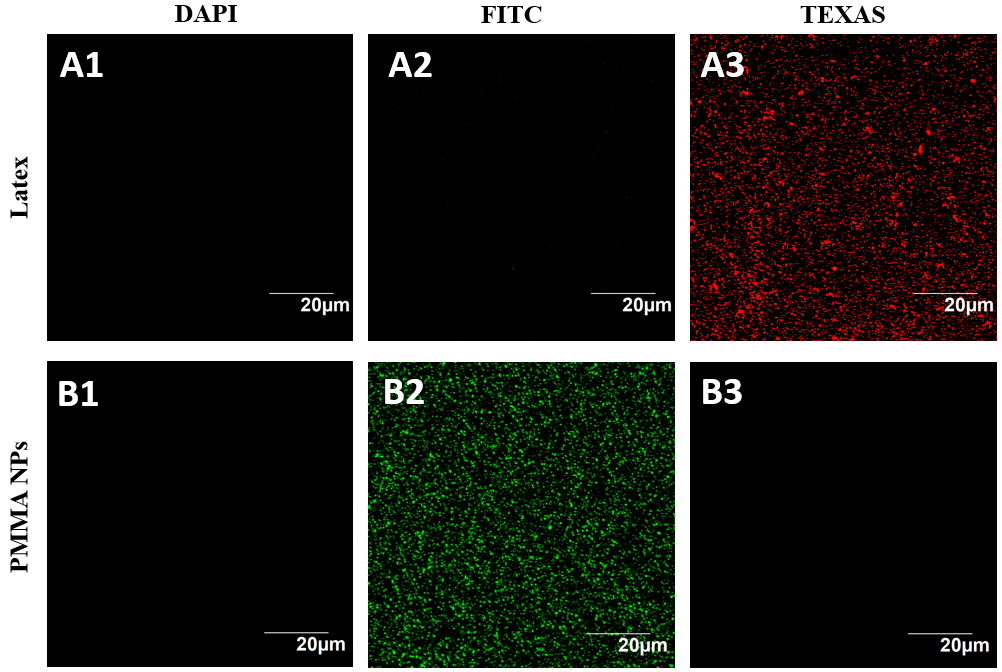


**Figure S7.** Confocal images of pure latex beads (A1 – A3) and PMMA NPs (B1 – B3, concertation 200 µg/ml) without any cells added. The samples were drop casted on glass slides, dried under ambient temperature and imaged using different channels of confocal microscopy. The scale bar is 20 µm.

8. Effect of polymer NPs on different cellular process

(i) Effect of NPs on intracellular ATP production


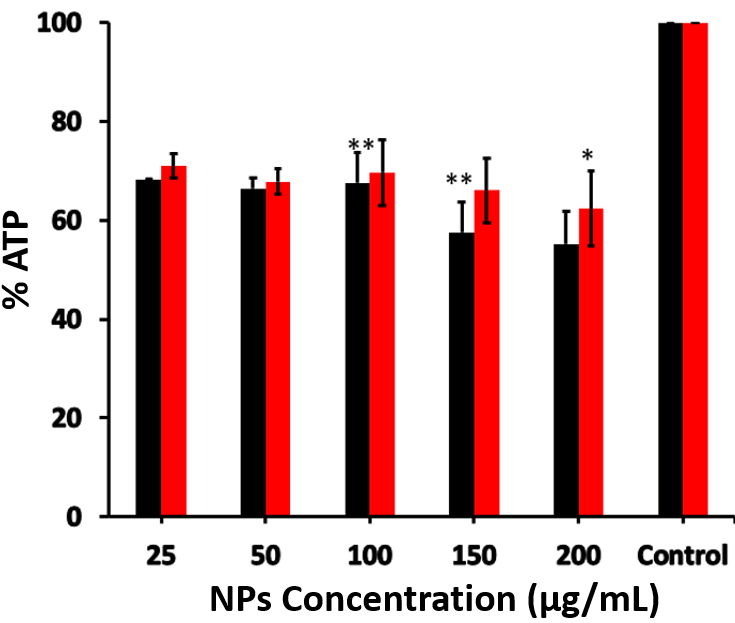


**Figure S8.** Intracellular ATP content of BHK-21 cells after exposure to PVC (◼) and PMMA (◼) NPs at different concentrations after 72 h. The results are given as % of the viability of the control and mean values of n = 3 independent experiments. Statistical analysis was done by one-way ANOVA (*p < 0.05, **p < 0.01, ***p < 0.001, ****p < 0.0001) and compared to control with no polymer NPs added.

(ii) Effect of polymer NPs on intracellular ROS production


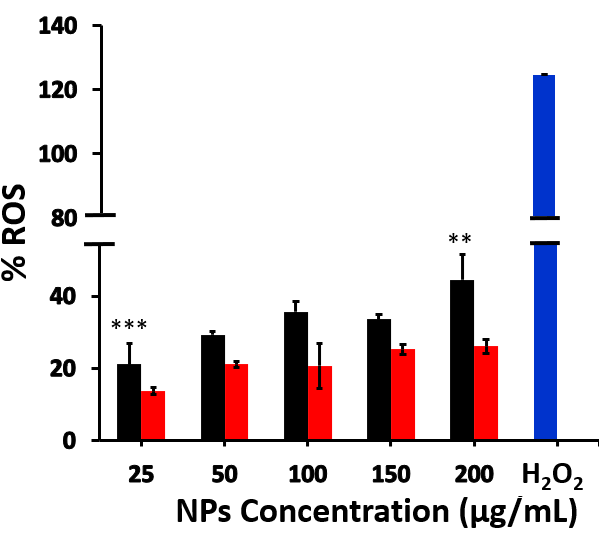


**Figure S9.** ROS production in BHK-21 cells exposed to PVC (◼) and PMMA (◼) nanoparticles at different concentrations after 72 h. The results are given as % of the viability of the control and mean values of n = 3 independent experiments. Statistical analysis was done by one-way ANOVA (*p < 0.05, **p < 0.01, ***p < 0.001, ****p < 0.0001) and compared with control with no polymer NPs added.

(iii) Effect of polymer NPs on intracellular LDH Release


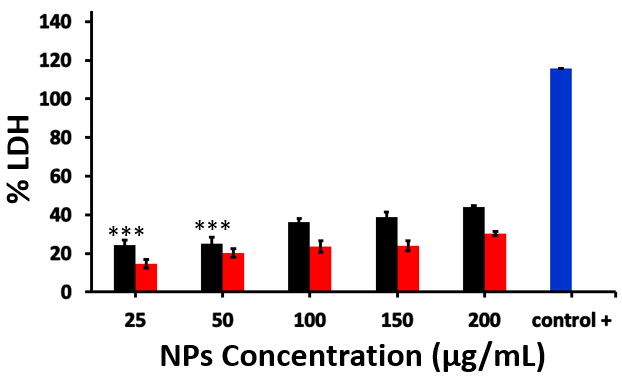


**Figure S10.** Concentration of LDH release into the medium after exposure to PVC (◼) and PMMA (◼) NPs at different concentrations after 72 h. The results are given as % of the viability of the control and mean values of n = 3 independent experiments. Statistical analysis was done by one-way ANOVA (*p < 0.05, **p < 0.01, ***p < 0.001, ****p < 0.0001) and compared with control with no polymer NPs added.
